# Supplementary material for: Mycoviral Diversity of Fusarium oxysporum f. sp. niveum in Three Major Watermelon-Production Areas in China
Source: Microorganisms. 2025 Apr 14;13(4):906. doi: 10.3390/microorganisms13040906 (PMC12029418; doi:10.3390/microorganisms13040906)
Supplement: Supplementary file 1 [file microorganisms-13-00906-s001.zip › microorganisms-3543517-supplementary.pdf]

SUPPLEMENTAL DOCUMENT

Table S1-S2; Figure S1-S2

Mycoviral Diversity of *Fusarium oxysporum* f. sp. *niveum* in Three Major Watermelon-Production Areas in China

Jiawang Yang [1,2,†], Yajiao Wang [1,†], Zihao Li [2], Sen Han [1], Bo Li [1,2,*] and Yuxing Wu [1,*]

[1] Institute of Plant Protection, Hebei Academy of Agricultural and Forestry Sciences, Baoding 071000, China; 16630393800@163.com (J.Y.); yajiaowang515@163.com (Y.W.); hansen19920603@163.com (S.H.)
[2] State Key Laboratory of North China Crop Improvement and Regulation, College of Plant Protection, Hebei Agricultural University, Baoding 071000, China; hbnylizihao@163.com
* Correspondence: zhibaolibo@163.com (B.L.); wyx1209@163.com (Y.W.)
† These authors contributed equally to this work.

Table S1. List of primers used in this study.

Name	Sequence (5'to3')	PCR product size	Contig name
FoNNAV1-S1-F	CCGTGGGAATGTTTGTC	962	contig58
FoNNAV1-S1-R	GCGGCAGCTAATGGTAC		
FoNNAV1-S2-F	TTGAGGGAGTTGGAAGT	965	
FoNNAV1-S2-R	CTCGGAGACGATTTGGTA		
FoNMV1-S1-F	TCGCATCCCTGTTGACTGAT	813	contig20
FoNMV1-S1-R	CGAGGGATGTGTGCAATTGT		
FoNMV1-S2-F	TGAAGGGTGAGGTGGAT	938	
FoNMV1-S2-R	AAATGCCGTTGGTAGAA		
FoNMTV1-S1-F	TTTACGACACCAGAGGGTTA	803	contig682
FoNMTV1-S1-R	GTATTATAGACGGCAAGGAA		
FoNMTV1-S2-F	TTCAGCAACAGATAGATTTC	911	
FoNMTV1-S2-R	TCGTATTATAGACGGCAAGG		
FoNMLV1-S1-F	ACATTCGGGTATAAGCCACA	966	contig453
FoNMLV1-S1-R	ATTCCATCCTTTCTCCCTGT		
FoNMLV1-S2-F	CGACCTAAAGGCCGCTAC	916	
FoNMLV1-S2-R	TTGCTGCTCTATTGATCCATAAC		
FoNTV1-S1-F	ATCCATGTAGTTCCACGCCA	834	contig164
FoNTV1-S1-R	ATCCATGTAGTTCCACGCCA		
FoNTV1-S2-F	CTGCGATATGCTCTGGT	811	
FoNTV1-S2-R	CTGTGGTTACGGGTGAA		
FoNCHV2-S1-F	AGACCGTTCGTTGATACTGA	773	contig89
FoNCHV2-S1-R	GCTTTCCCATTCTTGCTC		
FoNCHV2-S2-F	ATGACCGACCGAAGTAAA	753	
FoNCHV2-S2-R	CGTGGATGCCTTAGCAG		

Note: S1: The first pair of primers; S2: Second pair of primers.

Table S2. Molecular features of the mycoviruses identified in *Fusarium oxysporum* f. sp. *niveum*.

Contig name	Contig length (bp)	Virus name	Virus abbrev	Genome type	Virus family	identity (%)	Best hit	E value	Accession
contig177	3486	*Fusarium oxysporum* f. sp. *niveum* chrysovirus 1 dsRNA1	FoNCHV1	dsRNA	*Chrysoviridae*	99.5	Fusarium sacchari chrysovirus 1	0	QIQ28417.1
contig325	2790	*Fusarium oxysporum* f. sp. *niveum* chrysovirus 1 dsRNA2	FoNCHV1	dsRNA	*Chrysoviridae*	98.7	Fusarium sacchari chrysovirus 1	0	QIQ28419.1
contig407	2600	*Fusarium oxysporum* f. sp. *niveum* chrysovirus 1 dsRNA3	FoNCHV1	dsRNA	*Chrysoviridae*	99.3	Fusarium sacchari chrysovirus 1	0	QIQ28420.1
contig1649	1599	*Fusarium oxysporum* f. sp. *niveum* chrysovirus 1 dsRNA4	FoNCHV1	dsRNA	*Chrysoviridae*	94.3	Fusarium sacchari chrysovirus 1	0	QIQ28418.1
contig85	4418	*Fusarium oxysporum* f. sp. *niveum* chrysovirus 2 dsRNA1	FoNCHV2	dsRNA	*Chrysoviridae*	55.8	Ilyonectria pseudodestructans chrysovirus 1	2.04E-293	UVD33182.1
contig89	4342	*Fusarium oxysporum* f. sp. *niveum* chrysovirus 2 dsRNA2	FoNCHV2	dsRNA	*Chrysoviridae*	46.9	Ilyonectria pseudodestructans chrysovirus 1	1.39E-200	UVD33181.1
contig87	4378	*Fusarium oxysporum* f. sp. *niveum* chrysovirus 3	FoNCHV3	dsRNA	*Chrysoviridae*	93.9	Fusarium oxysporum chrysovirus 1	0	YP_009665200.1
contig11809	634	*Fusarium oxysporum* f. sp. *niveum* chrysovirus 4	FoNCHV4	dsRNA	*Chrysoviridae*	68.1	Pestalotiopsis theae chrysovirus 1	1.59E-20	QCY49460.1
contig164	3530	*Fusarium oxysporum* f. sp. *niveum* tick virus 1	FoNTV1	dsRNA	*Chrysoviridae*	85.4	Nanning Chrys tick virus 1	0	UYL95303.1
contig33373	260	*Fusarium oxysporum* f. sp. *niveum* Picobirnavirus 1	FoNPV1	dsRNA	*Picobirnaviridae*	92.6	Picobirnavirus sp.	1.98E-09	UJT31894.1
contig542	2544	*Fusarium oxysporum* f. sp. *niveum* Hadakavirus 1 dsRNA1	FoNHV1	ssRNA(+)	*Hadakaviridae*	97	Hadaka virus 1	0	YP_010840281.1
contig766	2297	*Fusarium oxysporum* f. sp. *niveum* Hadakavirus 1 dsRNA2	FoNHV1	ssRNA(+)	*Hadakaviridae*	99.3	Hadaka virus 1	0	YP_010840282.1
contig90	4313	*Fusarium oxysporum* f. sp. *niveum* Hadakavirus 1 dsRNA3	FoNHV1	ssRNA(+)	*Hadakaviridae*	98.5	Hadaka virus 1	0	YP_010840279.1
contig3627	1350	*Fusarium oxysporum* f. sp. *niveum* Hadakavirus 1 dsRNA4	FoNHV1	ssRNA(+)	*Hadakaviridae*	96.5	Hadaka virus 1	7.13E-164	YP_010840280.1
contig5119	1166	*Fusarium oxysporum* f. sp. *niveum* Hadakavirus 1 dsRNA5	FoNHV1	ssRNA(+)	*Hadakaviridae*	97	Hadaka virus 1	5.42E-150	YP_010840283.1
contig6291	1062	*Fusarium oxysporum* f. sp. *niveum* Hadakavirus 1 dsRNA6	FoNHV1	ssRNA(+)	*Hadakaviridae*	98.4	Hadaka virus 1	2.97E-165	YP_010840284.1
contig7055	1008	*Fusarium oxysporum* f. sp. *niveum* Hadakavirus 1 dsRNA7	FoNHV1	ssRNA(+)	*Hadakaviridae*	80.4	Hadaka virus 1	5.04E-76	YP_010840276.1
contig8759	908	*Fusarium oxysporum* f. sp. *niveum* Hadakavirus 1 dsRNA8	FoNHV1	ssRNA(+)	*Hadakaviridae*	91.7	Hadaka virus 1	2.00E-37	YP_010840274.1
contig9099	891	*Fusarium oxysporum* f. sp. *niveum* Hadakavirus 1 dsRNA9	FoNHV1	ssRNA(+)	*Hadakaviridae*	88.4	Hadaka virus 1	1.71E-91	YP_010840275.1
contig9161	888	*Fusarium oxysporum* f. sp. *niveum* Hadakavirus 1 dsRNA10	FoNHV1	ssRNA(+)	*Hadakaviridae*	90.1	Hadaka virus 1	6.72E-94	YP_010840277.1
contig682	2375	*Fusarium oxysporum* f. sp. *niveum* mitovirus 1	FoNMTV1	ssRNA(+)	*Mitoviridae*	64.1	Botrytis cinerea mitovirus 8	1.99E-302	QHJ68503.1
contig64489	275	*Fusarium oxysporum* f. sp. *niveum* mitovirus 2	FoNMTV2	ssRNA(+)	*Mitoviridae*	50	Henan forest mitovirus 6	8.49E-17	UPW42166.1
contig453	2256	*Fusarium oxysporum* f. sp. *niveum* mito-like virus 1	FoNMLV1	ssRNA(+)	*Mitoviridae*	70.5	Henan mito-like virus 31	0	UPW42176.1
contig64917	250	*Fusarium oxysporum* f. sp. *niveum* mito-like virus 2	FoNMLV2	ssRNA(+)	*Mitoviridae*	69.1	Xinjiang mito-like virus 58	5.65E-14	UPW42184.1

contig67309	246	*Fusarium oxysporum* f. sp. *niveum* splipalmivirus 1 RNA1	FoNSV1	ssRNA(+)	*Narnaviridae*	87.7	Oidiodendron maius splipalmivirus 1	9.67E-40	QNN89180.1
contig63076	253	*Fusarium oxysporum* f. sp. *niveum* splipalmivirus 1 RNA2	FoNSV1	ssRNA(+)	*Narnaviridae*	70.4	Oidiodendron maius splipalmivirus 1	1.20E-32	UJT31791.1
contig65881	271	*Fusarium oxysporum* f. sp. *niveum* narnavirus 2	FoNNV2	ssRNA(+)	*Narnaviridae*	90	Erysiphe necator associated narnavirus 49	9.04E-45	QJT93781.1
contig39542	327	*Fusarium oxysporum* f. sp. *niveum* Potyvirus 1	FoNPTV1	ssRNA(+)	*Potyviridae*	99.1	papaya ringspot virus	1.38E-60	KLP21678.1
contig20	7039	*Fusarium oxysporum* f. sp. *niveum* mymonavirus 1	FoNMV1	ssRNA(-)	*Mymonaviridae*	73.5	Plasmopara viticola lesion associated mymonavirus 1	0	YP_010798438.1
contig26	6714	*Fusarium oxysporum* f. sp. *niveum* mymonavirus 2	FoNMV2	ssRNA(-)	*Mymonaviridae*	29.1	Magnaporthe oryzae mymonavirus 1	1.48E-33	QVU39969.1
contig32	6457	*Fusarium oxysporum* f. sp. *niveum* mononegavirus1	FoNMGV1	ssRNA(-)	*Mymonaviridae*	46.2	Plasmopara viticola lesion associated mononegaambi virus 8	0	YP_010798440.1
contig58	5119	*Fusarium oxysporum* f. sp. *niveum* RNA virus 1	FoNNAV1	ssRNA(-)	-	52.8	Grapevine-associated negative single-stranded RNA virus 1	5.65E-104	QXN75409.1
contig27793	399	*Fusarium oxysporum* f. sp. *niveum* densovirus 1	FoNDV1	ssDNA(+/-)	*Parvoviridae*	100	Sitobion miscanthi densovirus	1.90E-36	AWS20450.1
contig44208	307	*Fusarium oxysporum* f. sp. *niveum* Capripoxvirus 1	FoNCPV1	dsDNA	*Poxviridae*	100	lumpy skin disease virusvirion core protein (lumpy skin disease virus), partial [Stenotrophomonas maltophilia]	5.28E-54	PTT44258.1
contig69281	248	*Fusarium oxysporum* f. sp. *niveum* leukemia virus 1	FoNLV1	ssRNA-RT	*Retroviridae*	100	Murine leukemia virus	1.22E-28	AAA46516.1
contig74422	253	*Fusarium oxysporum* f. sp. *niveum* Retrovirus 1	FoNRV1	ssRNA-RT	*Retroviridae*	100	Retrovirus-related Pol polyprotein [Yarrowia lipolytica]	2.27E-45	QNP98380.1
contig61628	282	*Fusarium oxysporum* f. sp. *niveum* Caulimovirus 1	FoNGV1	dsDNA-RT	*Caulimoviridae*	97.8	Caulimovirus viroplasmin-domain-containing protein [Fusarium solani]	1.72E-55	XP_046124817.1
contig425	2570	*Fusarium oxysporum* f. sp. *niveum* culmorum virus 1	FoNCV1	-	-	84.7	Fusarium culmorum virus 1	0	QIC51517.1
contig4624	951	*Fusarium oxysporum* f. sp. *niveum* narna-levi-like virus 1	FoNNLV1	-	-	98.9	XiangYun narna-levi-like virus 3	1.68E-120	UUG74238.1

Figure S1. Growth rate and spore yield of 150 FoN. (a) Growth rate of 150 FoN. Among them, 6 strains grew at a rate less than 0.8 cm per day, indicating abnormal morphology. (b) Spore yield of 150 FoN. Among them, the spore yield of 11 strains was less than 10×10^5, indicating abnormal morphology.

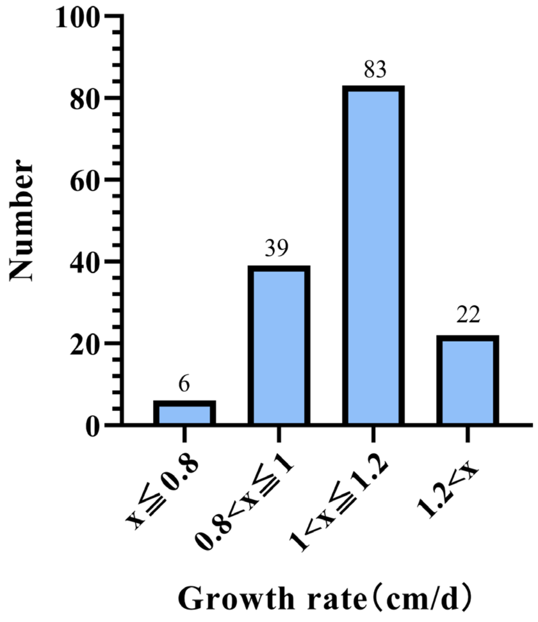

(a)

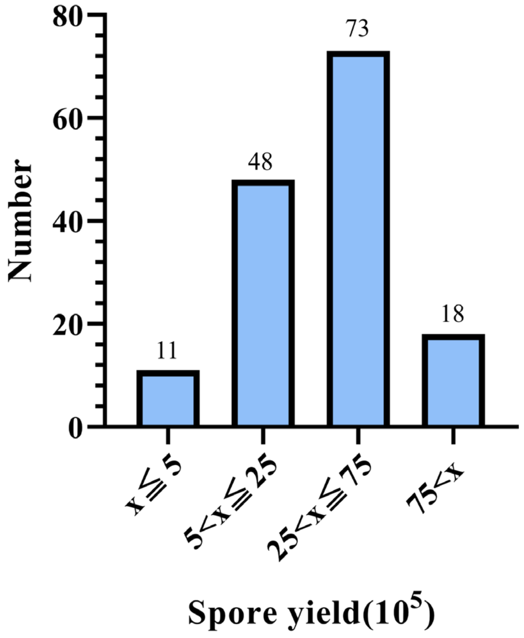

(b)

Figure S2. Strains with abnormal pigment production and colony morphology. (a) Strain with normal pigment production. (b-f) Five strains with abnormal pigment production. (G) Strain with normal colony morphology. (h-l) Five strains with abnormal colony morphology.

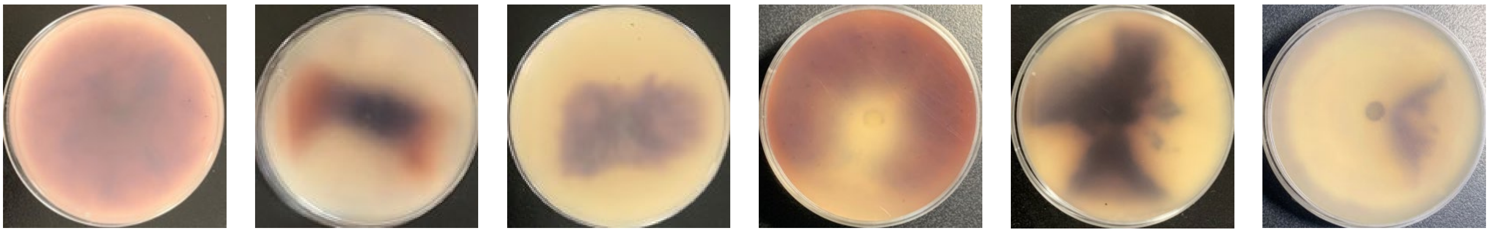

(a) (b) (c) (d) (e) (f)

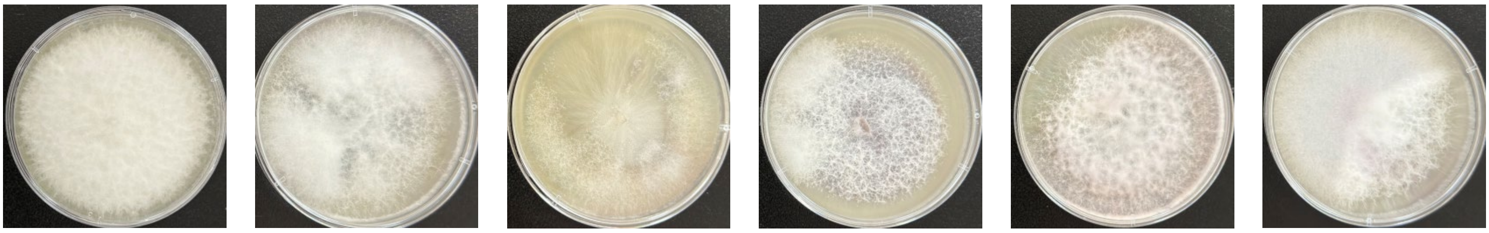

(g) (h) (i) (j) (k) (l)
